# Variable Number Tandem Repeats in the Mitochondrial DNA of *Lentinula edodes*

**DOI:** 10.3390/genes10070542

**Published:** 2019-07-17

**Authors:** Sinil Kim, Yelin Song, Byeongsuk Ha, Yoon Jung Moon, Minseek Kim, Hojin Ryu, Hyeon-Su Ro

**Affiliations:** 1 Division of Applied Life Science and Research Institute of Life Sciences, Gyeongsang National University, Jinju 52828, Korea; 2 Department of Biology, Chungbuk National University, Cheongju 28644, Korea

**Keywords:** *Lentinula edodes*, mating, mitochondrial DNA, tandem repeats, VNTR

## Abstract

Variable number tandem repeats (VNTRs) in mitochondrial DNA (mtDNA) of *Lentinula edodes* are of interest for their role in mtDNA variation and their application as genetic marker. Sequence analysis of three *L. edodes* mtDNAs revealed the presence of VNTRs of two categories. Type I VNTRs consist of two types of repeat units in a symmetric distribution, whereas Type II VNTRs contain tandemly arrayed repeats of 7- or 17-bp DNA sequences. The number of repeat units was variable depending on the mtDNA of different strains. Using the variations in VNTRs as a mitochondrial marker and the *A* mating type as a nuclear type marker, we demonstrated that one of the two nuclei in the donor dikaryon preferentially enters into the monokaryotic cytoplasm to establish a new dikaryon which still retains the mitochondria of the monokaryon in the individual mating. Interestingly, we found 6 VNTRs with newly added repeat units from the 22 mates, indicating that elongation of VNTRs occurs during replication of mtDNA. This, together with comparative analysis of the repeating pattern, enables us to propose a mechanistic model that explains the elongation of Type I VNTRs through reciprocal incorporation of basic repeat units, 5’-TCCCTTTAGGG-3’ and its complementary sequence (5’-CCCTAAAGGGA-3’).

## 1. Introduction

The mitochondrion is a key cellular organelle in eukaryotic organisms that plays a central role in the generation of adenosine triphosphate (ATP), the universal energy currency of living organisms. It is also involved in multiple cellular events, such as cell proliferation, differentiation, signaling, and apoptosis, through energy metabolism and reactive oxygen species generation [1,2,3,4] and as a provider of biosynthetic precursors [5,6]. It possesses a bacteria-like genome, suggesting its bacterial origin [7]. In the sexual reproduction of eukaryotic organisms, mitochondria are inherited from a single parent, mostly by maternal origin in the majority of animals and plants [8,9,10,11], while some coniferous plants show paternal inheritance [11]. 

Fungi show diverse features in mitochondrial inheritance. In the ascomycete yeast *Saccharomyces cerevisiae*, mitochondria of mating cells fuse to form a continuous reticulum with mixed mitochondrial DNAs (mtDNAs). The new diploid cell emerging from the mated cell can inherit mtDNA from a single parent or a mixture of mtDNAs from both parents depending on its budding sites [12,13]. The diploid cell having a mixture of mtDNAs eventually settles on a certain mitochondrial type through subsequent mitotic divisions [14]. Unlike ascomycete yeasts, basidiomycete yeasts, such as *Cryptococcus neoformans* and *Ustilago maydis*, show uniparental inheritance associated with certain mating types [15,16,17]. Genes involved in the mating process, such as the mating type-specific homeodomain genes *Sxi1a* and *Sxi2a* [18] and transcription factor *Mat2* in the mating pheromone response pathway [19], have been demonstrated to control the mitochondrial inheritance of *C. neoformans*. Depending on the mating strain pair, biparental inheritance also occurs, as shown in *C. gattii* [20]. 

The uniqueness of mitochondrial inheritance in the filamentous basidiomycetes comes from their mating behavior. Compatible mating in these fungi occurs when two monokaryotic mycelia of different mating types fuse (Mon–Mon mating). Generally, the nucleus from each monokaryon in the fused cell undergoes mitotic nuclear division, and the divided nuclei are transferred bilaterally to neighboring monokaryotic cells through clamp connections for the eventual establishment of dikaryons throughout the mycelia [21]. As a result, the Mon–Mon mating generates two different dikaryotic mates that have the same nuclear composition but different cytoplasmic contents when the dikaryons are isolated from the opposite edges of the growing mycelia. The mitochondria in the cytoplasm are therefore transmitted uniparentally, as shown in *Pleurotus ostreatus* [22], *Schizophyllum commune* [23], *Agaricus bitorquis* [24], *A. bisporus* [25], and *Coprinopsis cinerea* [26,27], with occasional mtDNA recombination in the dikaryons isolated at the junctional region of the mating [26,27].

Filamentous basidiomycetes can also perform mating between dikaryotic mycelia and monokaryotic mycelia (Di–Mon mating) to generate a new dikaryon, known as the Buller phenomenon [28,29]. In this process, one of the two types of nuclei in the dikaryon pairs with the nucleus from the monokaryon to establish a new dikaryon. For further understanding of this process, we approached the mobility of the nucleus and the inheritance of mtDNA in Di–Mon mating using *Lentinula edodes* as a model system, which is of interest in terms of the mating pheromone pathway [30], the nucleus specificity of gene expression [31], and the mating type diversity [32,33]. Particularly, the variable region in the *A* mating type genes, as a marker of each nucleus in the dikaryotic cytoplasm, was employed for the discrimination of each nucleus inside a dikaryon [32]. Together with the nuclear markers, mtDNA inheritance was monitored using variable number tandem repeats (VNTRs) in the mtDNAs of various strains of *L. edodes*. Various VNTRs are present in multiple copies in all eukaryotic mtDNAs and have previously been employed for identification purposes [34].

## 2. Materials and Methods 

### 2.1. Strains and Culture Conditions

The strains of *L. edodes* used, obtained from culture collections in Korea as previously described [32], are shown in Table S1. Mycelial culture was performed on potato dextrose agar (PDA; Oxoid, Hampshire, UK) or in potato dextrose broth (PDB; BD Difco, Sparks, MD) at 25 °C.

### 2.2. Mating between Dikaryons and Monokaryons

For Di–Mon mating, three to five pieces of agar blocks (0.5 × 0.5 cm) of a monokaryotic strain grown on PDA were placed on a new PDA plate (9 cm in diameter) in a semicircular arrangement, and the plate was incubated at 25 °C. After five days of incubation, a dikaryotic mycelial agar block (0.5 × 0.5 cm) was placed at the center of the semicircle where monokaryotic mycelia were propagated. The plate was further incubated for more than 10 days until the propagating dikaryotic mycelia completely mixed with the monokaryotic mycelia. The mated mycelial agar blocks were taken from the mycelial edges growing outward from the monokaryotic mycelial semicircle. 

### 2.3. Analysis of Mitochondrial DNA Sequences

The mitochondrial DNA sequence of *L. edodes* B-17 strain (Le-B17; [35]) and two published mtDNA sequences (GenBank accession numbers AB697988.1 and KY217797.1) available from a public database were compared by multiple sequence alignment using the MAFFT program (https://www.ebi.ac.uk/Tools/msa/mafft/). The lengths of mtDNAs Le-B17, AB697988.1, and KY217797.1 were 121,593 bp, 121,394 bp, and 116,819 bp, respectively. The variations among the three mtDNA sequences were identified manually from the multiple sequence alignment data. Tandem Repeats Finder [36] was employed for the analysis of variable number tandem repeats (VNTRs) within the mtDNAs. The mitochondrial gene arrangement of the *L. edodes* mtDNA was analyzed using the MFannot program at Robert Cedergren Centre, Université de Montréal (http://megasun.bch.umontreal.ca/cgi-bin/mfannot/mfannotInterface.pl). 

### 2.4. PCR Analyses Targeting VNTRs

Mycelia of *L. edodes* strains grown on PDA at 25 °C for seven days were collected, and the mycelia were ground with a mortar and pestle in liquid nitrogen. Total DNA was extracted using a genomic DNA extraction kit (HiGene Genomic DNA Prep kit; BIOFACT, Daejeon, Korea). The isolated total DNAs were subjected to PCR with specific primer sets (Table S2), targeting 200–500 bp regions containing target VNTRs to analyze VNTR markers within the mtDNAs. PCRs were conducted using a PCR premix (Maxime PCR premix kit; iNtRON Biotech, Eumseong, Korea) under the following conditions: first, denaturation at 95 °C for 5 min; followed by 30 cycles of denaturation at 95 °C for 30 s, annealing at 55 °C for 30 s, and polymerization at 72 °C for 30 s; and final incubation at 72 °C for 10 min. PCR products were analyzed by electrophoresis using 2% agarose gel. Sequences of the PCR products were determined after cloning into a TA cloning kit (pGEM-Teasy vector; Promega, Madison, WI, USA).

## 3. Results

### 3.1. VNTRs in the Mitochondrial DNA

**Figure 1:**
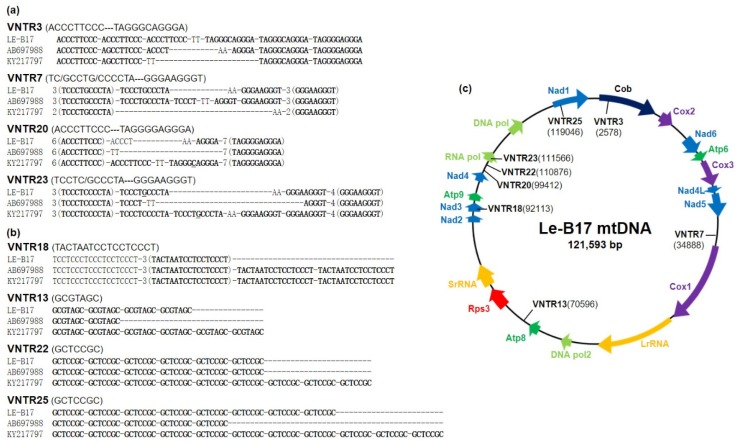
Variable number tandem repeats (VNTRs) in the mitochondrial DNA (mtDNA) of *Lentinula edodes*. (**a**) Type I VNTRs. VNTRs containing two different tandem repeats (TRs) from three different mtDNAs. Numbers in front of TRs indicate the numbers of repeating units; (**b**) Type II VNTRs. VNTRs containing single TRs. TRs are in boldface; (**c**) Arrangement of mitochondrial genes and the locations of TR markers in the mtDNA of *L. edodes* B17 strain.

Comparative analysis of the three *L. edodes* mtDNAs revealed 27 variable number tandem repeat (VNTR) regions (Figure S1). The VNTRs were largely categorized into two groups, depending on their repeating patterns. The Type I VNTRs contain two types of VNTR units in a symmetric distribution, including ACCCTTCCC-TAGGGSAGGGA (VNTR3 and VNTR20, where S stands for C or G), and TSCCTSCCCTA-GGGAAGGGT (VNTR7 and VNTR23), as shown in Figure 1a. Among the 27 VNTRs, 22 were within this group (Figure S1). An interesting feature of these VNTRs was the cross-complementarity of the repeat units. The ACCCTTCCC and TAGGGSAGGGA units in VNTR3 and VNTR20 are reverse-complementary to the GGGAAGGGT and TSCCTSCCCTA units in VNTR7 and VNTR23, respectively. 

Different from the Type I VNTRs, the Type II VNTRs consist of tandem repeats of GCTCCGC (VNTR10, VNTR22, and VNTR25), GCGTAGC (VNTR13), or TACTAATCCTCCTCCCT (VNTR18) (Figure 1b). The number of repeat units was variable depending on the mtDNAs. For example, the numbers of repeats of GCTCCGC units in VNTR25 were 8, 5, and 11 for Le-B17, AB697988.1, and KY217797.1, respectively. 

The VNTRs were distributed randomly throughout the mtDNA, mostly in the intergenic regions. Some VNTRs were found within the mitochondrial genes, such as VNTR3 in *COB*, VNTR18 in *NAD3*, VNTR23 in *RNAP*, and VNTR25 in *NAD1* (Figure 1c). However, all VNTRs in these genes located at the intronic regions (data not shown). 

### 3.2. VNTRs in the Verification of Mitochondria in *Lentinula edodes*

**Figure 2:**
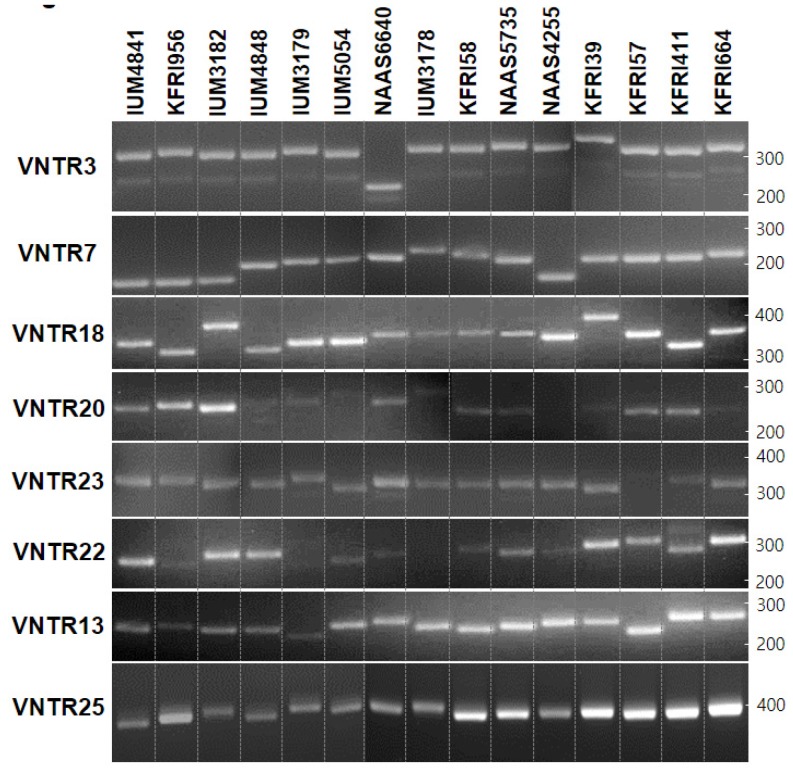
Length polymorphism of the VNTRs in the mtDNAs of different strains of *Lentinula edodes*.

In a previous study, verification of both nuclei in the various dikaryotic strains was enabled through simple PCR analyses of the variable sequence regions in the *A* mating type genes [32]. By a similar approach, the potential of VNTRs for the verification of mitochondria was explored in the various strains of *L. edodes*. First, sequence regions of mtDNA (200–500 bp in length) containing eight selected VNTRs were amplified using specific primer sets (Table S2). PCR analyses of the mtDNAs of 15 *L. edodes* strains showed variable length polymorphisms depending on the strain and VNTR (Figure 2, Table S3). VNTRs such as VNTR7, VNTR18, VNTR13, and VNTR25 were useful in the discrimination of mtDNAs in the different strains by yielding distinct DNA bands in variable lengths. For example, mtDNAs in the *L. edodes* strains IUM4841, KFRI956, and IUM3182 commonly had the same length VNTR7 (192 bp) but were discriminable by both length polymorphism in VNTR18 and copy number difference in VNTR25.

**Figure 3:**
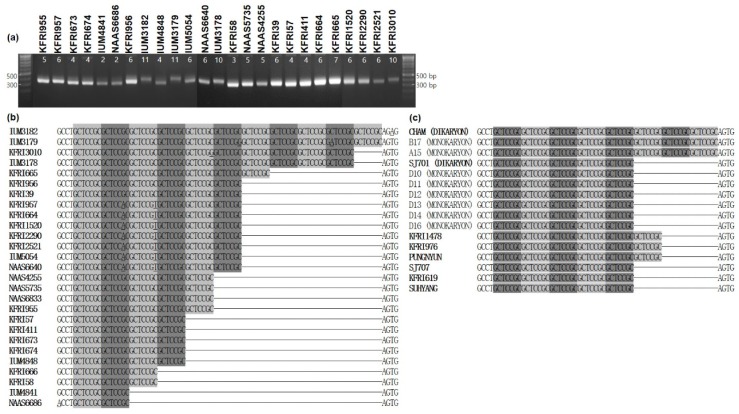
Analysis of VNTR25 in the mtDNAs of different strains of *Lentinula edodes*. (**a**) Length polymorphism of the PCR-amplified VNTR25 marker regions; (**b**) Repeat number variation in the sequences of VNTR25 from the wild strains. Single-nucleotide variations in the repeating unit are underlined; (**c**) Variations in the number of repeating units in VNTR25 of the selected cultivated strains.

VNTR polymorphism in mtDNA verification was further explored with regard to the repeat number variations in VNTR25 (GCTCCGC, Type II). The mtDNA regions containing VNTR25 (~400 bp) were amplified from 25 wild strains (Figure 3a), and the repeat numbers of the GCTCCGC motif were compared (Figure 3b). The repeat number was highly variable depending on the strain. The mtDNAs from the strains IUM3183 and IUM3179 carried the highest repeat number with 11 copies of the GCTCCGC motif, whereas those from the strains IUM4841 and NAAS6686 contained the lowest with two repeats. The former two strains were isolated from the same location—Mt. Seorak, Korea (Table S1). Interestingly, among the six-repeat strains, seven strains (KFRI957, KFRI665, KFRI1520, KFRI2290, KFRI2521, IUM5054, and NAAS6640) carried common single-nucleotide polymorphisms (SNPs) at the second (G→C) and third (C→T) motifs (Figure 3b). The strains KFRI957, KFRI1520, and NAAS6640 were isolated from the same location, Mt. Jumbong, while other SNP-carrying strains were from various parts of Korea (Table S1). KFRI956 isolated from Mt. Jumbong also carried six repeats but was devoid of SNPs.

The repeat number variation in the cultivated strains was less extreme. Some of the popular cultivated strains, including Cham, SJ701, KFRI1478, KFRI976, Pungnyun, SJ707, KFRI619, and Suhyang, have five, six, or eight repeat units. The repeat number in the mtDNA of dikaryons was consistently inherited by the monokaryons isolated from basidiospores of the dikaryons (Figure 3c). 

### 3.3. Unidirectional Movement of the Nucleus in Dikaryon–Monokaryon Mating

**Figure 4:**
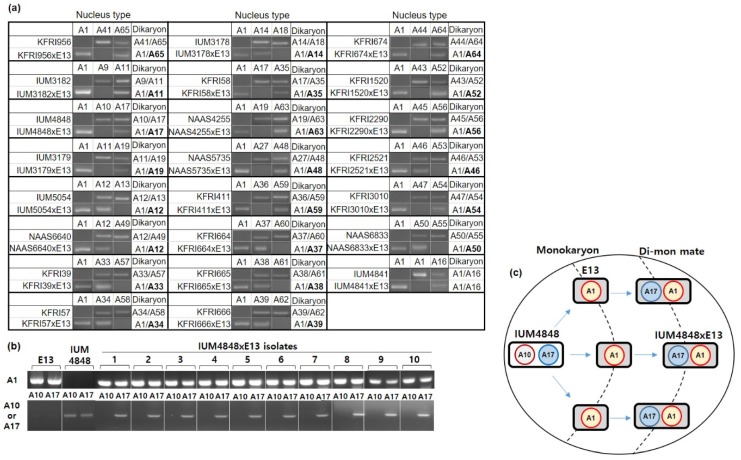
Unidirectional movement of the nucleus in Di–Mon mating. (**a**) Nuclear types in the mating partner dikaryon (e.g., KFRI956) and the resulting Di–Mon mate (e.g., KFRI956xE13). Di–Mon mating was performed between the partner dikaryons with different nuclear types and the monokaryon E13 with A1 nuclear type. The *A* mating types in the dikaryons were analyzed by PCR using specific primer sets. The two nuclear types in the dikaryons are summarized under the column “Dikaryon.” The nuclei in the Di–Mon mates received from the partner dikaryons are boldfaced; (**b**) Analysis of the nuclear types of 10 Di–Mon mates of IUM4848xE13 isolated from 10 different locations at the growing edge. PCR analyses were performed on the total DNA using primer sets targeting the *A1*, *A10*, or *A17* mating types; (**c**) Schematic presentation of the nucleus movement from IUM4848 to E13 to form IUM4848xE13. The donor dikaryon IUM4848 was placed at the left corner of the plate, and the receiving monokaryon E13 was placed at five locations in a semi-circle to surround the growing donor dikaryon. The Di–Mon mates were isolated at the outside of the growing monokaryon.

Di–Mon matings were performed between various dikaryotic strains and a monokaryotic E13 strain isolated from basidiospores of SJ701 [32]. The two nuclei in the dikaryotic strains and the nucleus in the E13 strain were identified by the *A* mating type gene marker developed previously [32]. The *A* mating type of the E13 strain was *A1*, and thus we recognized the nuclear type of this strain as an “A1” nucleus. Similarly, each of the two nuclei in any dikaryotic strain was identified with its *A* mating type (Figure 4a, Table 1). For example, the strain KFRI956 carried *A41* and *A65* mating types, representing the *A* mating types of the two nuclei, and thus we recognized each nucleus as “A41” or “A65.” The nuclear types of the new dikaryotic strains generated by Di–Mon mating together with the original dikaryotic strains are shown in Figure 4a. The Di–Mon mating of KFRI956 with E13 generated a new dikaryotic strain (KFRI956xE13) whose nuclear types are A1 and A65, indicating that the A65 nucleus, not the A42 nucleus, pairs with the A1 nucleus of E13. The Di–Mon matings in this experimental set revealed that the monokaryotic nucleus A1 always pairs with one of the two nuclei in the dikaryon to generate new dikaryotic strains with the nuclear types A1 and Ax, where Ax is the nuclear type of any nucleus in the original dikaryotic strain.

It appeared that there was no preference in the selection of the nucleus in the different Di–Mon mating pairs. The A11 nucleus in IUM3182 (A9/A11) paired with A1 in IUM3182x E13 (A1/A11). However, A11 was not chosen in IUM3179xE13 (A1/A19) where the nuclear type of IUM3179 was A11/A19 (Figure 4a). A similar feature was observed in the selection of A17 in IUM4848xE13 and KFRI58xE13 and of A19 in IUM3179xE13 and NAAS4255xE13. Only IUM5054xE13 (A1/A12) and NAAS6640xE13 (A1/A12) showed A12 preference. Overall, these data suggest that the pairing of nuclei in Di–Mon mating occurs between the monokaryotic nucleus and one of the two nuclei in the dikaryotic strain. 

We next asked whether nuclear selection is random or preferential in individual Di–Mon matings. For this, we isolated 10 Di–Mon mates of IUM4848xE13 from 10 different places in the same mating plate with IUM4848 (A10/A17) and E13 (A1). The PCR analysis revealed that all the Di–Mon isolates carried A1 and A17 nuclear types, indicating that the A1 nucleus paired exclusively with the A17 nucleus, not with A10 (Figure 4b,c). This was further confirmed by three independent mating analyses (Figure S2). The result suggests that nuclear selection is not random in the individual Di–Mon matings.

### 3.4. Mitochondrial Inheritance as a Marker of Cytoplasm Selection in Di–Mon Mating

**Figure 5:**
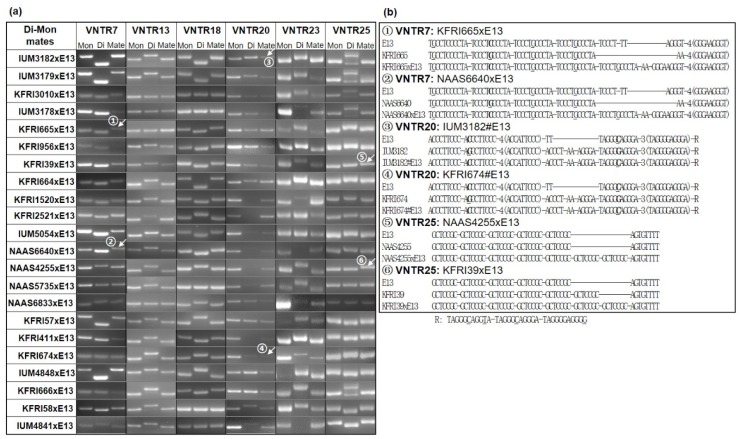
Mitochondrial inheritance in Di–Mon mating. (**a**) Length polymorphisms of VNTRs in the mtDNAs were investigated among dikaryotic strains (marked as “Di”), monokaryotic strain E13 (marked as “Mon”), and the Di–Mon mates (marked as “Mate”). The VNTRs with unexpected lengths are numbered; (**b**) Analyses of repeat sequences in VNTRs with unexpected lengths in the Di–Mon mating. SNP positions are boldfaced.

The VNTRs in the mtDNAs were employed for the verification of mitochondrial composition in Di–Mon mating. The length polymorphisms of six VNTRs (VNTR7, VNTR13, VNTR18, VNTR20, VNTR23, and VNTR25) were investigated to identify the origin of mitochondria in the mated dikaryons (see the column marked as “Mate” in Figure 5a) generated by Di–Mon matings between dikaryons (column marker as “Di”) and the monokaryotic E13 strain (marked as “Mon”) (Figure 5a). The results together with the nuclear types are summarized in Table 1. Most of the VNTR markers were able to distinguish the mitochondrial types in the original dikaryons (“Di”) from the E13 monokaryon (Figure 5a). For example, the mitochondrial type in IUM4841 was distinguishable from E13 in length polymorphism with all the VNTRs except for VNTR18. Moreover, analysis of the VNTR markers in the Di–Mon mate (“Mate”) tells that the mitochondria in IUM4841xE13 originate from the monokaryon E13 but not from the dikaryon IUM4841 (“Di”). In fact, it turned out that all the Di–Mon mates carry mitochondria from the monokaryon E13 (Figure 5a, Table 1). All things considered, it is highly possible that one of the nuclei in the dikaryotic strain randomly enters into the monokaryotic cytoplasm to establish a new dikaryon in Di–Mon mating.

### 3.5. Elongation of Repeat Units in VNTRs and Implications for mtDNA Evolution 

One interesting observation noted in the analysis of VNTRs in Di–Mon mating was the presence of slightly bigger VNTR bands in the Di–Mon mates than those expected from the monokaryon (Figure 5a, numbered and marked with arrows). Sequence determination on the VNTR markers revealed that the VNTR7s in the Di–Mon mates KFRI665xE13 and NAAS6640xE13 were lengthened by half of the repeat unit (GCCCTA) in the left repeat (TCCTGCCCTA) and half (GGGA) in the right repeat (GGGAAGGGT) compared to that in the monokaryotic E13 (Figure 5b). Nonetheless, the VNTR7s were determined to have originated from the monokaryon (E13) because of the presence of a common SNP in the second repeat unit in the left repeat where E13 and the Di–Mon mates carry “C” while the partner dikaryons (KFRI665 or NAAS6640) carry “G” at this position (Figure 5b). More clear evidence of VNTR elongation was found in the analysis of VNTR25s, in which both the Di–Mon mates NAAS4255xE13 and KFRI39xE13 were found to have two additional repeats of GCTCCGC (Figure 5b). Different from the above two VNTRs, the VNTR20s in IUM3182xE13 and KFRI674xE13 showed DNA bands of the same sizes as those of the original dikaryons IUM3182 and KFRI673, respectively, implying their origins from the mating dikaryons (Figure 5b, marked by arrows in VNTR20). However, the presence of two common SNPs in the VNTR20s in the monokaryon and the Di–Mon mates suggests that the VNTR20s in the Di–Mon mates were generated by the VNTR20 of the monokaryon with the addition of half repeats, similar to the elongation of VNTR7 in Di–Mon mates.

## 4. Discussion

Mitochondrial inheritance in the filamentous basidiomycetes is distinct from that in other organisms due to the lack of nuclear and cytoplasmic fusions during mating [21]. Only the nucleus from a mating cell (in the mycelium) enters the cytoplasmic space of the partner cell to establish a new dikaryotic mycelial cell. Because this process occurs reciprocally in both monokaryons in Mon–Mon mating, the new dikaryon can have one of the two cytoplasms, thus having one of the two mitochondrial types depending on the parental monokaryon that receives the incoming nucleus [22,23,24,25,26,27]. However, biparental mitochondrial inheritance has also been reported in the zone of contact between two monokaryotic strains [20,26,27,37,38]. In the present study on the Di–Mon mating of *L. edodes*, our results suggest that a nucleus from the dikaryon unidirectionally enters into the monokaryotic cytoplasm in the newly generated dikaryotic cell and that one of the two nuclei within the mating partner dikaryotic cell is preferentially selected to establish a new dikaryon (Figure 4).

We discovered 27 VNTRs that consist of either two types of tandem repeats (Type I) or variable numbers of direct repeats (Type II) in the mtDNA of *L. edodes*. Type I VNTRs, consisting of two types of VNTR units in a symmetric distribution, are distinct from other VNTRs. A long stretch of (ACCCTTCCC)n-(TAGGGSAGGGA)n in VNTR3 and VNTR20 forms inverted repeats with (TCCCTSCCCTA)n-(GGGAAGGGT)n in VNTR7 and VNTR23. Type II VNTRs found in *L. edodes* are two 7 bp tandem repeats of GCTCCGC and GCGTAGC and a 17 bp repeat of TACTAATCCTCCTCCCT (Figure 1b). This type of VNTR is also found in *P. ostreatus*, a representative edible mushroom, as (CTGCTATG)n and (CTGCTACG)n [39]. Different from the Type II VNTRs in *L. edodes*. However, the two direct repeats in *P. ostreatus* form inverted repeats with (CATAGCAG)n and (CGTAGCAG)n, respectively, similar to the Type I VNTRs in *L. edodes*. 

Variations in the number of tandem repeats are thought to be generated by genetic recombination [40,41,42] and/or slipped-strand mispairing in the mtDNA [43,44]. The tandem repeats in animals and plants have been reported to be involved in mtDNA stability by insertion and deletion of the repeat array [45,46]. In the fungal kingdom, the repeats function in mitochondrial gene rearrangement mostly through recombination and contribute to mtDNA evolution among various fungal classes [47]. 

**Figure 6:**
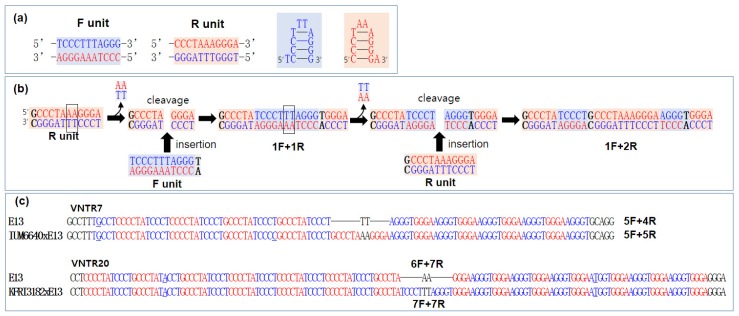
Proposed model for the elongation of Type I VNTRs. (**a**) The basic sequence units in the Type I VNTR. (**b**) Elongation of the VNTR through the incorporation of the sequence units. The F unit and the R unit can be incorporated into the R unit (left) and the F unit (right), respectively, after double-strand break and removal of the dinucleotide AA or TT (boxed). Additional nucleotides incorporated together with the F or R unit are black-colored. (**c**) Analyses of repeats in VNTR7 and VNTR20. The numbers in front of F or R (e.g., 5F+4R) indicate the numbers of the incorporated F or R units in each VNTR.

Our VNTR analyses show that the number of tandem repeats is highly variable among different strains of *L. edodes* (Figure 3) and that occasional addition of new repeating units can occur during mtDNA replication for both Type I and Type II VNTRs (Figure 5). Accordingly, comparative examination of the Type I VNTRs reveals that the VNTRs in this group expand by alternate addition of TCCCT and G/CCCCTA in the left side and AGGGT and GGGA in the right side of the VNTR. Although the detailed factors involved in this process remain elusive, we have come up with a mechanistic idea that explains the elongation process. There are two basic sequence units in Type I VNTRs: 5’-TCCCTTTAGGG-3’ (F unit) and its complementary sequence unit, 5’-CCCTAAAGGGA-3’ (R unit) (Figure 6a). Both of the units can form hairpin structures as shown in Figure 6a. Elongation of the VNTR occurs when the F unit together with an additional T at the 3’-end incorporates into the center of the R unit after cleavage and removal of the dinucleotide AA (Figure 6b, left panel). The R unit also incorporates into the F unit after removal of the dinucleotide TT at the center with an additional G or C at the 5’-end of the R unit. Repeated and alternate addition of F and R units can generate a long stretch of VNTR containing an alternating motif of TCCT and G/CCCCTA at the left side and GGGA and AGGGT at the right side (Figure S3). This scheme can explain the elongation process and the length polymorphisms of all Type I VNTRs. For example, the VNTR7s in the E13 and IUM6640xE13 differ in the number of repeat units. E13 contains five F units and four R units, whereas IUM6640xE13 carries five F units and five R units, indicating the addition of a new R unit during mtDNA replication (Figure 6c). Similarly, the VNTR20 in the Di–Mon mate KFRI3182xE13 carries one additional F unit to that in the E13 mtDNA. It is notable that the unit close to the central TT or AA is the newly incorporated repeat unit. 

## 5. Conclusions

The present study demonstrated that the VNTRs in the mtDNA of *L. edodes* are highly variable depending on the strain, and these variations can be employed for the verification of mitochondrial type in different strains. It is suggested that the VNTRs (Type I) are elongated through the repeated and alternate incorporation of repeating units at the center of the VNTRs. Through the analyses of the VNTRs and the *A* mating type, which represent the mitochondrial type and the nuclear type, respectively, it is also suggested that one of the two nuclei inside the dikaryon moves to the monokaryotic cytoplasm to establish a new dikaryon that retains its mitochondria originating from the monokaryon in the Di–Mon mating. From a practical viewpoint, the VNTR markers in this study can be highly valuable tools for strain development when they are used together with nuclear markers such as *A* mating type markers [32] and simple sequence repeat markers [48].

## Figures and Tables

**Table 1:** Analyses of the nuclear and mitochondrial types of Di–Mon mates.

Strain Number	Nuclear Type			Mitochondrial Type in the Di–Mon Mate^a^						
	Di	Mon	Mate	VNTR7	VNTR13	VNTR18	VNTR20	VNTR23	VNTR25	Overall
IUM3182	A9/A11	A1	A1/A11	Mon	Mon	Mon	**Mon** ^b^	Mon	Mon	Mon
IUM3179	A11/A19		A1/A19	Mon	Mon	Mon	Mon	Mon	Mon	Mon
KFRI3010	A47/A54		A1/A54	Mon	ND^c^	ND	ND	Mon	Mon	Mon
IUM3178	A14/A18		A1/A14	Mon	ND	ND	ND	Mon	Mon	Mon
KFRI665	A38/A61		A1/A38	**UP** ^d^	ND	Mon	ND	Mon	Mon	Mon
KFRI956	A41/A65		A1/A65	Mon	Mon	Mon	ND	Mon	Mon	Mon
KFRI39	A33/A57		A1/A33	Mon	Mon	Mon	ND	Mon	**UP**	Mon
KFRI664	A37/A60		A1/A37	Mon	Mon	Mon	Mon	Mon	Mon	Mon
KFRI1520	A43/A52		A1/A52	Mon	Mon	Mon	Mon	Mon	Mon	Mon
KFRI2521	A46/A53		A1/A46	Mon	Mon	Mon	Mon	ND	Mon	Mon
IUM5054	A12/A13		A1/A12	Mon	Mon	Mon	Mon	ND	Mon	Mon
NAAS6640	A12/A49		A1/A12	**UP**	Mon	Mon	Mon	Mon	Mon	Mon
NAAS4255	A19/A63		A1/A63	Mon	Mon	Mon	Mon	Mon	**UP**	Mon
NAAS5735	A27/A48		A1/A48	ND	Mon	ND	Mon	Mon	ND	Mon
NAAS6833	A50/A55		A1/A50	Mon	ND	Mon	ND	Mon	ND	Mon
KFRI57	A34/A58		A1/A34	Mon	Mon	Mon	ND	Mon	ND	Mon
KFRI411	A36/A59		A1/A59	Mon	Mon	Mon	Mon	Mon	Mon	Mon
KFRI674	A44/A64		A1/A64	ND	Mon	Mon	**Mon** ^b^	Mon	Mon	Mon
IUM4848	A10/A17		A1/A17	Mon	Mon	Mon	ND	Mon	Mon	Mon
KFRI666	A39/A62		A1/A39	ND	Mon	Mon	ND	Mon	Mon	Mon
KFRI58	A17/A35		A1/A35	Mon	Mon	ND	Mon	Mon	Mon	Mon
IUM4841	A1/A16		A1/A16	Mon	Mon	ND	Mon	Mon	Mon	Mon

^a^ The mitochondrial origin in the Di–Mon mates was investigated by determining the length polymorphisms of VNTRs. “Mon” indicates that the mitochondria are from the monokaryon (E13). ^b^ The two boldfaced “Mon” contained the same length in VNTR20 as the dikaryotic strains but carried the same single-nucleotide polymorphisms (SNPs) as the monokaryotic strain. ^c^ ND, not differentiable. ^d^ UP, VNTRs in the Di–Mon mate were bigger than those in the monokaryon or the dikaryons.

## Supplementary Materials

The following are available online at https://www.mdpi.com/2073-4425/10/7/542/s1, Figure S1: VNTRs in the mtDNAs of *Lentinula edodes*; Figure S2: Analysis of nuclear types of Di-Mon mates of IUM4848xE13 from three independent matings, Figure S3: Elongation of Type I VNTR through reciprocal incorporation of repeating units, Table S1: Strains used in this study, Table S2: Primers for VNTR analysis used in this study, Table S3: Analysis of VNTR length polymorphism in different VNTRs.
