# Supplementary material for: Variable Number Tandem Repeats in the Mitochondrial DNA of Lentinula edodes
Source: Genes (Basel). 2019 Jul 17;10(7):542. doi: 10.3390/genes10070542 (PMC6679062; doi:10.3390/genes10070542)
Supplement: Supplementary file 1 [file genes-10-00542-s001.pdf]

Supplementary Materials: Variable Number Tandem Repeats in the Mitochondrial DNA of *Lentinula edodes*

```
VNTR1  Le_B17    295   AGCCTTCCCAGCCTTCCCAGCCTTCCCACCCTTCCCACCCTTCCCACCCTTCCCTTT-----------AGGGCAGGGATAGGGGAGGGATAGGGGAGGGATAGGGGAGGGATAGGGGAGGGATAGGGGAGGCACTACCT  422
       AB697988        AGCCTTCCCAGCCTTCCCACCCTTCCCACCCTTCCCACCCTTCCCACCCTTCCCTTT-----------AGGGCAGGGATAGGGGAGGGATAGGGGAGGGATAGGGGAGGGATAGGGGAGGGATAGGGGAGGCACTACCT
       KY217797        AGCCTTCCCAGCCTTCCCACCCTTCCCACCCTTCCCACCCTTCCCACCCTTCCCTTTAGGGCAGGGATAGGGGAGGGATAGGGCAGGGATAGGGGAGGGATAGGGGAGGGATAGGGGAGGGATAGGGGAGGCACTACCT

VNTR2  Le_B17    1366  TCCCTCCCCTAAAGGGAAGGGTGGGAAGTGTGGGAAGGGTGGGAAGGGTGGGAAGGGTGGGTAGG  1430
       AB697988        TCCCTCCCCTAAAGGGAAGGGTGGGAAGTGTGGGAAGGGTGGGAAGGGTGGGAAGGGTGGGTAGG
       KY217797        TCCCTGCCCTAAAGGGAAGGGT---------GGGAAGGGTGGGAAGGGTGGGAAGGGTGGGTAGG

VNTR3  Le_B17    2734  ACCCTTCCCACCCTTCCCACCCTTCCCTTTAGGGCAGGGATAGGGCAGGGATAGGGGAGGGATAGCCACTGCTCCCCTCCCACCCTAAAGG  2825
       AB697988        ACCCTTCCCAGCCTTCCCACCCT----------AAAGGGATAGGGCAGGGATAGGGGAGGGATAGCCACTGCTCCCCTCCCACCCTAAAGG
       KY217797        ACCCTTCCCAGCCTTCCCTT--------------------TAGGGCAGGGATAGGGCAGGGATAGCCACTGCTCCCCTCCCACCCTAAAGG

VNTR4  Le_B17    9332  ACCCTTCCCACCCTTCCCACCCTTCCCACCCTTCCCACCCTTCCCACCCT-----------AAAGGGATAGGGCAGGGATAGGGCAGGGATAGGGCAGGGATAGGGCAGGGATAGGGGAGGCACTTCC  9448
       AB697988        ACCCTTCCCACCCTTCCCACCCTTCCCACCCTTCCCACCCT---------AAAGGGATAGGGCAGGGATAGGGCAGGGATAGGGCAGGGATAGGGCAGGGATAGGGCAGGGATAGGGGAGGCACTTCC
       KY217797        ACCCTTCCCACCCTTCCCACCCTTCCCACCCTTCCCACCCTTCCCTTT---------------------AGGGCAGGGATAGGGGAGGGATAGGGCAGGGATAGGGCAGGGATAGGGGAGGCACTTCC

VNTR5  Le_B17    18469 TCCCTCCCCTATCCCTCCCCTATCCAGCCTATTCCACCCTATCCCACCCTATCCCTCCCCTATCCCTGCCCTAAAGGGAAGGGTGGGAAGGGTGGGAAGGGTGGGAAGGGTGCTGGGTGGAATAGGGAAGG  18599
       AB697988        TCCCTCCCCTATCCCTCCCCTATCCAGCCTATTCCACCCTATCCCACCCTATCCCTCCCCTATCCCTGCCCTAAAGGGAAGGGTGGGAAGGGTGGGAAGGGTGGGAAGGGTGCTGGGTGGAATAGGGAAGG
       KY217797        TCCCTCCCCTATCCCTCCCCTATCCAGCCTATTCCACCCTATCCCACCCTATCCCTCCCCTATCCCTTT----------AGGGTGGGAAGGGTGGGAAGGGTGGGAAGGGTGCTGGGTGGAATAGGGAAGG

VNTR6  Le_B17    20581 CTTTCCCCCTCCCTTCTCCCTGCCCTCCCCTATCCCAGCCTTCCCACCCTTCCCAGCCT---------------------------AAAGGGATAGGGAGGGATAGGGCTGGGATAGGGATATGGAGGGTGGCTAGGGTGGGGGG  20745
       AB697988        CTTTCCCCCTCCCTTCTCCCTGCCCTCCCCTATCCCAGCCTTCCCACCCTTCCCAGCCT---------------------------AAAGGGATAGGGAGGGATAGGGCTGGGATAGGGATATGGAGGGTGGCTAGGGTGGGGGG
       KY217797        CTTTCCCCCTCCCTTCTCCCTGCCCTCCCCTATCCCAACCTTCCCACCCT---------AAAGGGATAGGGAGGGATAGGGCTGGGATAGGGATATGGAGGGATAGGGCTGGGATAGGGATATGGAGGGTGGCTAGGGTGGGGGG

VNTR7  Le_B17    34864 AGGCTGGGTGCCTTTGCCTCCCCTATCCCTGCCCTATCCCTGCCCTATCCCTGCCCTA----------AAGGGAAGGGTGGGAAGGGTGGGAAGGGTGGGAAGGGTGCAGGGTGCTCCTT  34973
       AB697988        AGGCTGGGTGCCTTTGCCTCCCCTATCCCTCCCCTATCCCTGCCCTATCCCTGCCCTATCCCTTTAGGGTGGGAAGGGTGGGAAGGGTGGGAAGGGTGGGAAGGGTGCAGGGTGCTCCTT
       KY217797        AGGCTGGGTGGCTATCCCTCCCCTATCCCTGCCCTA--------------------------------------------------AAGGGAAGGGTGGGAAGGGTGCAGGGTGCTCCTT

VNTR8  Le_B17    35366 TATAACCAAAATAGAATTAGAATTATAAGCAGGCAGCCTTCCC---------ACCCTTCCCACCCTAAAGGGATAGGGCAGGGATAGGGGAGGCATCCATACGACGGGGATGTTTAGTGT  35476
       AB697988        TATAACCAAAATAGAATTAGAATTATAAGCAGGCAGCCTTCCCACCCTTCCCACCCTTCCCACCCTAAAGGGATAGGGCAGGGATAGGGGAGGCATCCATACGACGGGGATGTTTAGTGT
       KY217797        TATAACCAAAATAGAATTAGAATTATAAGCAGGCAGCCTTCCC---------ACCCTTCCCACCCTAAAGGGATAGGGCAGGGATAGGGGAGGCATCCATACGACGGGGATGTTTAGTGT

VNTR9  Le_B17    47883 TTTGATGAAGCCTTCCCTCCCTA--------------------AAGGGAAGGATTACGAGAAAAATGACAGTCAAAA  47940
       AB697988        TTTGATGAAGCCTTCCCTCCCTATCCCTGCCCTAAAGGGAAGGGTGGGAAGGATTACGAGAAAAATGACAGTCAAAA
       KY217797        TTTGATGAAGCCTTCCCTCCCTA--------------------AAGGGAAGGATTACGAGAAAAATGACAGTCAAAA

VNTR10 Le_B17    49625 GGCGGGCTGCAGCGTAGCGCTCCGCGCTCCGCGCTCCGCAGAGTTTTTTT  49674
       AB697988        GGCGGGCTGCAGCGTAGCGCTCCGCGCTCCGCGCTCCGCAGAGTTTTTTT
       KY217797        GGCGGGCTGCAGCGTAGCGCTCCGC--------------AGAGTTTTTTT

VNTR11 Le_B17    58676 ACTATACCTATCCCTCCCCTATCCCTCCCCTATCCCTTTAGGCTGGGAAGGGTGGGTGGTTATCCCTTT  58744
       AB697988        ACTATACCTATCCCTCCCCTATCCCTCCCCTATCCCTTTAGGCTGGGAAGGGTGGGTGGTTATCCCTTT
       KY217797        ACTATACCTATACCTCCCCTATCCCTGCCCTA----------AAGGGAAGGCTGGGTGGTTATCCCTTT
```

```
VNTR12 Le_B17    68844 TCCCTCCCCTATCCCTGCCCTATCCCTGCCCTATCCCTGCCCTA-----------------------------AAGGGAAGGGTGGGAAGGGTGGGAAGGGTGGGTGCCTTAGGCTGG 68932
       AB697988        TCCCTCCCCTATCCCTGCCCTATCCCTGCCCTATCCCTGCCCTA-----------------------------AAGGGAAGGGTGGGAAGGGTGGGAAGGGTGGGTGCCTTAGGCTGG
       KY217797        TCCCTGCCCTATCCCTGCCCTATCCCTGCCCTATCCCTCCCCTATCCCTGCCCTAAAGGGAAGGGTGGGAAGGGTGGGAAGGGTGGGAAGGGTGGGAAGGGTGGGTGCCTTAGGCTGG

VNTR13 Le_B17    70656 GGCTCATCCCTGCGTAGCGCGTAGCGCGTAGCGCGTAGC--------------CTTCGCACCAAA 70706
       AB697988        GGCTCATCCCTGCGTAGCGCGTAGC----------------------------CTTCGCACCAAA
       KY217797        GGCTCATCCCTGCGTAGCGCGTAGCGCGTAGCGCGTAGCGCGTAGCGCGTAGCCTTCGCACCAAA

VNTR14 Le_B17    70923 GGCCCCCTCCCCTATCCCTGCCCTATCCCTCCCCTATCCCTGCCCTATCCCTGCCCTATCCCTGCCCTA----------AAGGGAAGGGTGGGAAGGGTGGGAAGGGTGGGAAGGGTGGGTGGCT 71037
       AB697988        GGCCCCCTCCCCTATCCCTGCCCTATCCCTCCCCTATCCCTGCCCTATCCCTGCCCTATCCCTGCCCTATCCCTTTAGGGTGGGAAGGGTGGGAAGGGTGGGAAGGGTGGGAAGGGTGGGTGGCT
       KY217797        GGCCCCCTCCCCTATCCCTGCCCTATCCCTGCCCTATCCCTGCCCTATCCCTGCCCTATCCCTGCCCTA----------AAGGGAAGGGTGGGAAGGGTGGGAAGGGTGGGAAGGGTGGGTGGCT

VNTR15 Le_B17    85572 TCCCAGCCCTATCCCTCCCCTATCCCTGCCCTATCCCTTT----------AGGGTGGGAAGGGTGGGATAGGGGAGGGTAAGGGTGCTGGG 85652
       AB697988        TCCCAGCCCTATCCCTCCCCTATCCCTGCCCTATCCCTTT----------AGGGTGGGAAGGGTGGGATAGGGGAGGGTAAGGGTGCTGGG
       KY217797        TCCCAGCCCTATCCCTCCCCTATCCCTGCCCTATCCCTGCCCTAAAGGGAAGGGTGGGAAGGGTGGGATAGGGGAGGGTAAGGGTGCTGGG

VNTR16 Le_B17    86658 AGTGCCTTTCCCACCCTTCCCTCCCCTATCCCTTT----------AGGGTGGGAAGGGTTGGAAAGGGTGGAGGGGGGGG 86727
       AB697988        AGTGCCTTTCCCACCCTTCCCTCCCCTATCCCTTT----------AGGGTGGGAAGGGTTGGAAAGGGTGGAGGGGGGGG
       KY217797        AGTGCCTTTCCCACCCTTCCCTCCCCTATCCCTCCCCTAAAGGGAAGGGTGGGAAGGGTTGGAAAGGGTGGAGGGGGGGG

VNTR17 Le_B17    88676 TCCCTCCCCTATCCCTGCCCTATCCCTGCCCTATCCCTGCCCTATCCCTGCCCTAGCCCTTT-----------------------------------------------------------------------------AGGGTGGGAAGGCGGA 88753
       AB697988        TCCCTCCCCTATCCCTGCCCTATCCCTGCCCTATCCCTGCCCTATCCCTGCCCTAGCCCTTT-----------------------AGGGTGGGAAGGGTGGGAAGGGTGGGAAGGGTGGGAAGGGTGGGAGCCTAAGGCTGGGTGGGAAGGCGGA
       KY217797        TCCCACCCCTATCCCTCCCCTATCCCTCCCCTATCCCTCCCCTATCCCTCCCCTATCCCTCCCCTATCCCTCCCCTATCCCTGCCCTAAAGGGAAGGGTGGGAAGGGTGGGAAGGGTGGGAAGGGTGGGAGCCTAAGGCTGGGTGGGAAGGCGGA

VNTR18 Le_B17    92225 TCCTCCCTCCCTCCTCCCTTACTAATCCTCCTCCCTTACTAATCCTCCTCCCTTACTAATCCTCCTCCCT----------------------------------ACGTAGGATGGG 92306
       AB697988        TCCTCCCTCCCTCCTCCCTTACTAATCCTCCTCCCTTACTAATCCTCCTCCCTTACTAATCCTCCTCCCTTACTAATCCTCCTCCCTTACTAATCCTCCTCCCTACGTAGGATGGG
       KY217797        TCCTCCCTCCCTCCTCCCTTACTAATCCTCCTCCCTTACTAATCCTCCTCCCTTACTAATCCTCCTCCCTTACTAATCCTCCTCCCTTACTAATCCTCCTCCCTACGTAGGATGGG

VNTR19 Le_B17    97430 TCCCGCCTTCCCACCCTTCCCACCCTTCCCACCCTTCCCTTTAGGGCAGGGATAGGGGAGGGATAGGGGAGGGATAGGGGAGGCACTGCACCCTAAAGGG 97529
       AB697988        TCCCGCCTTCCCACCCTTCCCACCCTTCCCACCCTTCCCTTTAGGGCAGGGATAGGGGAGGGATAGGGGAGGGATAGGGGAGGCACTGCACCCTAAAGGG
       KY217797        TCCCGCCTTCCCACCCTTCCCACCCTTCCCTT--------------------TAGGGCAGGGATAGGGGAGGGATAGGGGAGGCACTGCACCCTAAAGGG

VNTR20 Le_B17    99502 TCCCTCCCACCCTTCCCACCCTTCCCACCATTCCCACCCTTCCCACCCTTCCCACCCTTCCCACCCT----------AAAGGGATAGGGCAGGGATAGGGGAGGGATAGGGGAGGGATAGGGGAGGGATAGGGCAGG 99628
       AB697988        TCCCTCCCACCCTTCCCACCCTTCCCACCATTCCCACCCTTCCCACCCTTCCCACCCTTCCCTT--------------------TAGGGCAGGGATAGGGGAGGGATAGGGGAGGGATAGGGGAGGGATAGGGCAGG
       KY217797        TCCCTCCCACCCTTCCCACCCTTCCCACCATTCCCACCCTTCCCACCCTTCCCACCCTTCCCACCCTTCCCTTTAGGGCAGGGATAGGGGAGGGATAGGGCAGGGATAGGGGAGGGAAAGGGGAGGGATAGGGCAGG
.
VNTR21 Le_B17   106853 CCTTCCCTCCCCTCCCACCCTTCCCACCCT----------AAAGGGATAGGGGAGGGATAGGTAAGGGAGGGGAGGG 106919
       AB697988        CCTTCCCTCCCCTCCCACCCTTCCCACCCT----------AAAGGGATAGGGGAGGGATAGGTAAGGGAGGGGAGGG
       KY217797        CCTTCCCTCCCCTCCCACCCTTCCCACCCTTCCCTTTAGGGCAGGGATAGGGGAGGGATAGGTAAGGGAGGGGAGGG

VNTR22 Le_B17   110906 GTTTGTCGCTCCGCGCTCCGCGCTCCGCGCTCCGCGCTCCGCGCTCCGC---------------------AGTGTTTT 110962
       AB697988        GTTTGTCGCTCCGCGCTCCGCGCTCCGCGCTCCGCGCTCCGCGCTCCGC---------------------AGTGTTTT
       KY217797        GTTTGTCGCTCCGCGCTCCGCGCTCCGCGCTCCGCGCTCCGCGCTCCGCGCTCCGCGCTCCGCGCTCCGCAGTGTTTT

VNTR23 Le_B17   111683 TCCCTGCCCTATCCCTCCCCTATCCCTCCCCTATCCCTGCCCTA--------------------AAGGGAAGGGTGGGAAGGGTGGGAAGGGTGGGAAGGGTGGGAAGGGTTGAGTG 111779
       AB697988        TCCCTGCCCTATCCCTCCCCTATCCCTCCCCTATCCCTTT------------------------------AGGGTGGGAAGGGTGGGAAGGGTGGGAAGGGTGGGAAGGGTTGAGTG
       KY217797        TCCCTCCCCTATCCCTCCCCTATCCCTCCCCTATCCCTCCCCTATCCCTGCCCTAAAGGGAAGGGTGGGAAGGGTGGGAAGGGTGGGAAGGGTGGGAAGGGTGGGAAGGGTTGAGTG

VNTR24 Le_B17   115902 ACCCTTCCCACCCTTCCCACCCTTCCCACCCTTCCCACCCTTCCCACCCTTCCCTTTAGGGCAGGGATAGGGGAGGGATAGGGCAGGGATAGGGGAGGGATAGGGCAGGGATAGGGGAGGGATAGCCACCCCCCC 116036
```

```
        AB697988        ACCCTTCCCACCCTTCCCACCCTTCCCACCCTTCCCACCCTTCCCACCCTTCCCTTTAGGGCAGGGATAGGGCAGGGATAGGGCAGGGATAGGGCAGGGATAGGGCAGGGATAGGGGAGGGATAGCCACCCCCCC
        KY217797        ACCCTTCCCACCCTTCCCACCCTTCCCACCCTTCCCACCCTTCCCTT--------------------TAGGGCAGGGATAGGGGAGGGATAGGGGAGGGATAGGGGAGGGATAGGGGAGGGATAGCCACCCCCCC

VNTR25  Le_B17   119220 GCCTGCTCCGCGCTCCGCGCTCCGCGCTCCGCGCTCCGCGCTCCGCGCTCCGCGCTCCGC---------------------AGTGTTTTAAATCCT 119294
        AB697988        GCCTGCTCCGCGCTCCGCGCTCCGCGCTCCGCGCTCCGC------------------------------------------AGTGTTTTAAATCCT
        KY217797        GCCTGCTCCGCGCTCCGCGCTCCGCGCTCCGCGCTCCGCGCTCCGCGCTCCGCGCTCCGCGCTCCGCGCTCCGCGCTCCGCAGTGTTTTAAATCCT

VNTR26  Le_B17   120015 CCTATCCCACCCCTCCCCTATCCCAGCCTCCCCTATCCCTGCCCTATCCCTC-CCCTA----------AAGGGAAGGGTGGGAAGGGTGGGAAGGCTGGGTAGGGTGGGTAGGCAGGGT 120122
        AB697988        CCTATCCCACCCCTCCCCTATCCCAGCCTCCCCTATCCCTGCCCTATCCCCTGCCCTA----------AAGGGAAGGGTGGGAAGGGTGGGAAGGCTGGGTAGGGTGGGTAGGCAGGGT
        KY217797        CCTATCCCACCCCTCCCCTATCCCAGCCTCCCCTATCCCTGCCCTATCCCTC-CCCTATCCCTTTAGGGTGGGAAGGGTGGGAAGGGTGGGAAGGCTGGGTAGGGTGGGTAGGCAGGGT

VNTR27  Le_B17   120844 CCCAGCCCTATCCCTTCCCTATCCCTCCCCTATCCCTCCCCTATCCCTGCCCTA--------------------AAGGGAAGGGTGGGAAGGGTGGGAAGGGTGGAAAGGCTGGGTGGCT 120943
        AB697988        CCCAGCCCTATCCCTTCCCTATCCCTCCCCTATCCCTCCCCTATCCCTGCCCTA--------------------AAGGGAAGGGTGGGAAGGGTGGGAAGGGTGGAAAGGCTGGGTGGCT
        KY217797        CCCAGCCCTATCCCGCCCCTATCCCTGCCCTATCCCTCCCCTATCCCTGCCCTATCCCTGCCCTAAAGGGAAGGGTGGGAAGGGTGGGAAGGGTGGGAAGGGTGGGAAGGCTGGGTGGCT
```

Figure S1. VNTRs in the mtDNAs of *Lentinula edodes*.

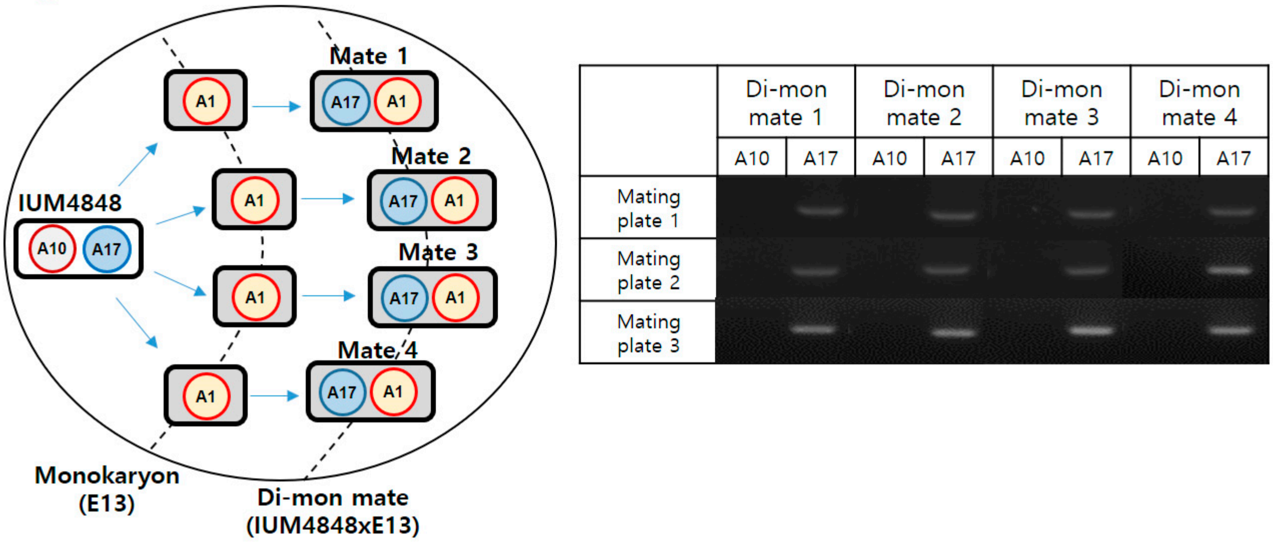

Figure S2. Analysis of nuclear types of Di-Mon mates of IUM4848xE13 from three independent matings.

Fig. S3

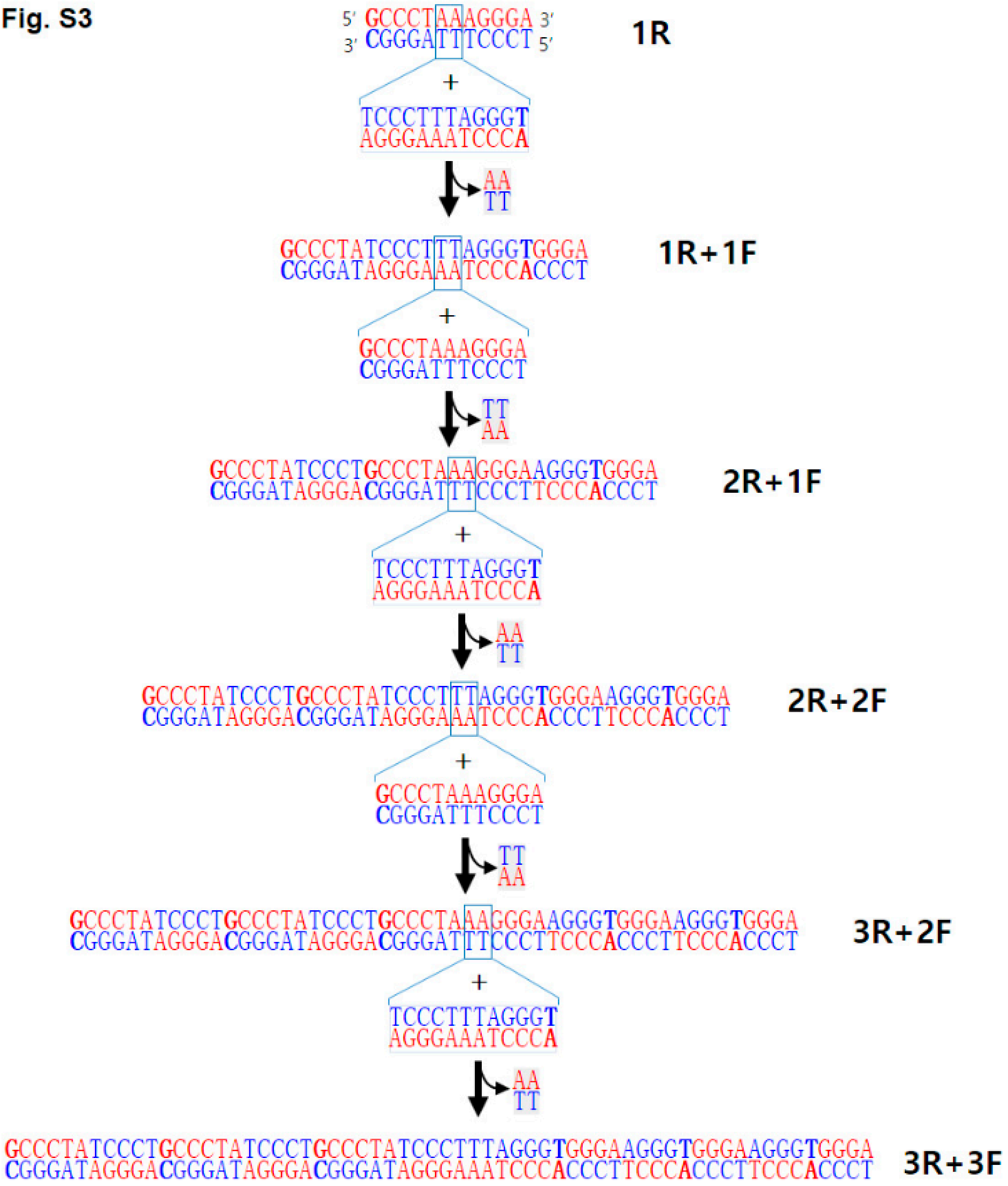

Figure S3. Elongation of Type I VNTR through reciprocal incorporation of repeating units.

Table S1. Strains used in this study.

	Strain number	Mating types		Collection		VNTR25 repeat number*
		A mating type	B mating type	Site	Year	
Wild strains	IUM3182	A9/A11	B7/B9	Mt. Seorak		11
	IUM3179	A11/A19	B7/B9	Mt. Seorak		11
	KFRI3010	A47/A54	B1/B15 or B3/B13	Mt. Hwaak	2015	10
	IUM3178	A14/A18	B3/B11	Mt. Seorak		10
	KFRI665	A38/A61	B8/B15 or B9/B14	Mt. Gyebang	2004	7
	KFRI956	A41/A65	B6/ND	Mt. Jumbong	2007	6
	KFRI39	A33/A57	B1/B15 or B3/B13	Mt. Jiri	1983	6
	KFRI957	A32/A42	B2/B15 or B3/B14	Mt. Jumbong	2007	6
	KFRI664	A37/A60	B4/ND	Mt. Gyebang	2004	6
	KFRI1520	A43/A52	B3/B6	Mt. Jumbong	2011	6
	KFRI2290	A45/A56	B13/B15	Mt. Kariwang	2013	6
	KFRI2521	A46/A53	B7/B9	Mt. Seorak	2013	6
	IUM5054	A12/A13	B4/ND	Mt Deogyu		6
	NAAS6640	A12/A49	B10/B11	Mt. Jumbong	2013	6
	NAAS4255	A19/A63	B4/B5	Mt. Odae	1994	5
	NAAS5735	A27/A48	B1/B3	Mt. Seorak	2010	5
	NAAS6833	A50/A55	B4/B15 or B6/B13	Mt. Seorak	2014	5
	KFRI955	A1/A36	B7/B13	Mt. Jumbong	2007	5
	KFRI57	A34/A58	B1/B3	Mt. Jiri	1984	4
	KFRI411	A36/A59	B8/B12 or B9/B11	Mt. Seorak	1999	4
	KFRI673	A40/A51	B1/B8 or B2/B7	Mt. Gyebang	2004	4
	KFRI2101	A44/A64	B8/B15 or B9/B14	Mt. Odae	2013	4
	IUM4848	A10/A17	B14/B15	Mt. Halla		4
	KFRI666	A39/A62	B8/B15 or B9/B14	Mt. Gyebang	2004	3
	KFRI58	A17/A35	B8/B14/or B9/B13	Mt. Jiri	1984	3
	IUM4841	A1/A16	B7/B14 or B8/B13	Mt. Jiri		2
	NAAS6686	A1/A30	B1/B8 or B2/B7	Mt. Jumbong	2013	2
Cultivated strains	CHAM	A1/A5	B11/B12			8
	SJ701	A1/A5	B4/B12			5
	KFRI1478	A1/A8	B2/B9			6
	KFRI976	A1/A4	B8/B13			6

Pungnyung	A1/A7	B2/B12	6
SJ707	A5/A7	B4/B12	5
KFRI619	A3/A7	B4/B12	5
Suhyanggo	A1/A7	B2/B12	5

*The repeat number in the VNTR25 are summarized based on the sequence analyses shown in Figures 3(B) and 3(C).

Table S2. Primers for VNTR analysis used in this study.

	Forward	Reverse
VNTR3	GAGACTACACATGGAGCTAGATTTAATC	CACCCTCAGCCTAGCCACTATC
VNTR7	CTCCTGCAGTTGCCCTCCCCTAT	AATGGAGGCGAGTCGAACACC
VNTR13	CCTCGCCTTTGTAAAAGTA	GAACAGCCGCAGGTTTTGC
VNTR18	GCGTCTCCTACGCGTCTCCTAC	CACCCTCAGCCTAGCCACTATC
VNTR20	GGAAAGGCACTGCACCCTAGC	CCTGACTTTCCTTTTTCCAAGTG
VNTR22	GGTAATAACCTGCAACCTTCC	CTTAAACCACTCAGCCATAGAAC
VNTR23	GGAGGCTAGGTATCACTTCCCCGTG	ACTGCACTTAGTTTGGCGAGCTG
VNTR25	GAGGTAAGGTCGTTCTTC	CAAATAGGCCTAAAGGAG

Table S3. Analysis of VNTR length polymorphism in different VNTRs.

Strain number	Mitochondrial repeat length polymorphism (bp)						
	**VNTR3**	**VNTR7**	**VNTR18**	**VNTR20**	**VNTR23**	**VNTR13**	**VNTR25***
IUM4841	300	192	350	275	350	256	2
KFRI956	320	192	306	285	350	263	6
IUM3182	300	192	383	275	340	249	11
IUM4848	300	202	306	285	350	256	4
IUM3179	320	232	350	285	370	235	11
IUM5054	300	232	350	295	340	263	6
NAAS6640	200	232	360	285	350	270	6
IUM3178	320	242	360	295	350	263	10
KFRI58	320	232	360	275	350	256	3
NAAS5735	340	202	360	275	350	263	5
NAAS4255	320	192	350	ND	350	270	5
KFRI39	360	202	383	285	340	270	6
KFRI57	320	202	350	275	370	249	4
KFRI411	320	202	306	275	370	277	4
KFRI664	340	232	350	285	350	277	6

*Numbers in this column represent the numbers of GCTCCGC repeats in the mtDNA.

© 2019 by the authors. Submitted for possible open access publication under the terms and conditions of the Creative Commons Attribution (CC BY) license (http://creativecommons.org/licenses/by/4.0/).
